# Evaluating the role of age on speech-in-noise perception based primarily on temporal envelope information

**DOI:** 10.1016/j.heares.2025.109236

**Published:** 2025-03-07

**Authors:** Jonathan Regev, Andrew J. Oxenham, Helia Relaño-Iborra, Johannes Zaar, Torsten Dau

**Affiliations:** a Hearing Systems Section, Department of Health Technology, Technical University of Denmark, 2800 Kgs. Lyngby, Denmark; b Auditory Perception and Cognition Laboratory, Department of Psychology, University of Minnesota, MN, 55455 Minneapolis, USA; c Eriksholm Research Centre, 3070 Snekkersten, Denmark; d Department of Biomedical Engineering, University of Rochester, NY, 14642 Rochester, USA

**Keywords:** Ageing, Temporal envelope perception, Speech perception, Amplitude modulation processing, Amplitude modulation masking

## Abstract

Acoustic amplitude modulation (AM) patterns carry important information, particularly in speech. AM masking, influenced by frequency selectivity in the modulation domain, is considered a crucial factor for speech intelligibility in noisy environments. Based on recent evidence suggesting an age-related decline in AM frequency selectivity, this study investigated whether increased AM masking in older listeners is associated with reduced speech intelligibility. Speech reception thresholds (SRTs) were measured using tone-vocoded speech and maskers with no modulation, broadband AM, or narrowband AM at varying modulation frequencies. AM masked thresholds were assessed for a 4-Hz target modulation frequency. The study included young (*N* = 14, 19–25 years) and older (*N* = 14, 57–79 years) listeners with normal hearing. It was hypothesized that SRTs would be higher for the older group with modulated maskers and that the age-related increase in SRT would depend on the masker’s modulation frequency content. The speech intelligibility results showed that maskers with broadband AM produced higher SRTs than unmodulated maskers. However, SRTs varied little with masker-modulation center frequency across the range tested (2–32 Hz). While older listeners exhibited lower AM frequency selectivity than young listeners, they did not consistently exhibit higher SRTs than their young counterparts across maskers. However, there was a trend for the effect of age to be greater for maskers with broadband AM than for unmodulated maskers. Overall, despite supportive trends, the results do not conclusively demonstrate that older listeners are more susceptible than young listeners to AM masking of speech.

## 1. Introduction

The auditory system exhibits acute sensitivity to envelope fluctuations in sound, commonly referred to as amplitude modulation (AM). This sensitivity has been extensively documented in numerous perceptual studies (e.g., Viemeister, 1979; Dau et al., 1997; Kohlrausch et al., 2000) and physiological investigations (e.g., Langner and Schreiner, 1988; Langner, 1992; see Joris et al., 2004 for a comprehensive review). Patterns of AM in speech are critical for intelligibility, as they convey substantial information (Houtgast and Steeneken, 1985; Smith et al., 2002; Elliott and Theunissen, 2009). Indeed, envelope fluctuations in just a few frequency regions are sufficient for accurate speech recognition, even when spectral cues are significantly reduced (Shannon et al., 1995; Dorman et al., 1997; Xu et al., 2005). Conversely, restricting the envelope frequencies conveyed by speech impairs speech recognition (Drullman et al., 1994a, 1994b; Arai et al., 1999; Xu et al., 2005; Apoux and Bacon, 2008a; Elliott and Theunissen, 2009).

Given the importance of AM for speech intelligibility, AM masking has been proposed as a critical factor influencing speech comprehension in noise (Dubbelboer and Houtgast, 2008; Jørgensen and Dau, 2011; Stone et al., 2011, 2012; Jørgensen et al., 2013, 2015; Stone and Moore, 2014). Similar to masking in the audio-frequency domain, AM masking refers to the reduced detectability of a target AM in the presence of masker AM. Evidence for AM masking has been provided by several perceptual studies (Bacon and Grantham, 1989; Houtgast, 1989; Dau et al., 1997, 1999; Ewert and Dau, 2000; Ewert et al., 2002; Regev et al., 2023a) and recent neurophysiological research (Viswanathan et al., 2021). The concept that AM masking affects the intelligibility of speech in noise has led to the hypothesis that performance is linked to the signal-to-noise ratio in the modulation domain (SNR_env_; Dubbelboer and Houtgast, 2008; Jørgensen and Dau, 2011; Jørgensen et al., 2013, 2015). Empirical support for this role of AM masking in speech intelligibility comes from studies using noise types with varying intrinsic envelope content to mask filtered speech (Stone et al., 2012; Stone and Moore, 2014) or tone-vocoded speech (Stone et al., 2011; Oxenham and Kreft, 2014, 2016). Further evidence arises from research that directly manipulates the SNR_env_ by attenuating noise modulations (Wójcicki and Loizou, 2012; Jørgensen et al., 2015).

Behavioral studies on AM masking have demonstrated the frequency-selective nature of AM perception, often modelled as a modulation filterbank (Dau et al., 1997, 1999; Ewert and Dau, 2000). This model is based on the assumption that stimulus AM is spectrally decomposed via a series of bandpass modulation filters. Computational speech-intelligibility models incorporating this concept – using either the SNR_env_ (Jørgensen and Dau, 2011; Jørgensen et al., 2013) or envelope-based correlation measures (Relaño-Iborra et al., 2016, 2019; Zaar and Dau, 2017; Zaar et al., 2017; Steinmetzger et al., 2019) at the output of the modulation filters – have proven highly effective. Overall, AM frequency selectivity, as represented by these modulation filters, has been identified as crucial for predicting speech intelligibility (see Relaño-Iborra and Dau, 2022 for a review).

Regev et al. (2023a) recently reported an age-related reduction in AM frequency selectivity when comparing young and older listeners with normal hearing (hereafter referred to as NH listeners). This reduction may partly contribute to the well-documented decline in speech perception in noise with age (CHABA, 1988; Pichora-Fuller and Souza, 2003; Füllgrabe et al., 2015; Goossens et al., 2017; Decruy et al., 2019). The findings of Regev et al. (2023b) support the existence of such a link, suggesting that a measure of AM frequency selectivity may help explain variations in speech intelligibility beyond the effects of age and audibility. However, these results were obtained by pooling data from young and older NH listeners, as well as older listeners with hearing impairment. As such, they did not definitively demonstrate that an age-related reduction in AM frequency selectivity is directly associated with poorer speech intelligibility. A direct test of this hypothesis would require a comparison of intelligibility scores between young and older NH listeners under conditions where speech intelligibility primarily depends on envelope cues.

One such study compared small groups of young (*N* = 4) and older (*N* = 9) NH listeners in their ability to understand tone-vocoded speech in the presence of a spectrally matched masker (Oxenham and Kreft, 2016). The masker either lacked amplitude fluctuations (equivalent to adding a DC component to the speech envelope in each vocoder channel) or contained fluctuations similar to those produced by a Gaussian noise masker. The results indicated no strong differences in intelligibility scores between young and older listeners. However, due to the small sample size, no definite conclusions could be drawn about the specific interaction between age and AM masking in speech. In addition, because the modulation masker was derived from broadband noise, it was not possible to assess the importance of specific modulation frequencies in explaining potential differences in speech masking between young and older listeners.

Several studies have investigated the importance of specific modulation frequencies for speech intelligibility. Acoustic analyses have shown that the syllable rate of natural speech (Greenberg et al., 2003; Giraud and Poeppel, 2012; Ding et al., 2017; Varnet et al., 2017) is approximately 4 Hz. This rate corresponds to the peak in speech modulation power when processed through a modulation filterbank that mimics human AM frequency selectivity (Houtgast and Steeneken, 1985; Schlueter et al., 2014; Varnet et al., 2017). Further studies manipulating modulation energy have provided evidence for the critical role of low modulation frequencies for speech intelligibility. Research removing specific modulation frequencies from speech (e.g., Drullman et al., 1994a, 1994b; Arai et al., 1999; Xu et al., 2005; Apoux and Bacon, 2008a) or adding masker modulations to speech (Apoux and Bacon, 2008b; Stone and Moore, 2014) typically highlights modulation frequencies between about 1 and 16 Hz as essential. Some studies have found that frequencies between 1 and 7 Hz (Elliott and Theunissen, 2009) or below 8 Hz (Drullman et al., 1994b; Arai et al., 1999) are especially important. Thus, based on both acoustic analyses and perceptual studies, it appears that modulation frequencies within a 2- to 4-octave band centered around 4 Hz are the most critical for speech intelligibility.

The aim of this study was to assess whether age-related declines in AM frequency selectivity (as postulated by Regev et al., 2023a) are associated with poorer speech intelligibility for older listeners. Specifically, the study tested whether older listeners are more susceptible to AM masking and whether reduced AM frequency selectivity with age is associated with differences between young and older NH listeners in the AM-frequency-selective effects observed in a speech masking task. To address these questions, tone vocoding, similar to the method used by Oxenham and Kreft (2014, 2016), was employed so that the processed speech would convey primarily envelope cues. Speech reception thresholds (SRTs) were measured using maskers modulated with narrowband-noise AM, centered at modulation frequencies ranging from 2 to 32 Hz in octave steps. Two reference maskers were also included: (1) a vocoded speech-shaped noise (SSN) with broadband envelope fluctuations, and (2) an unmodulated complex tone (CT) lacking inherent modulations. In addition to the intelligibility of masked speech, AM frequency selectivity was measured using 4-Hz sinusoidal target AM imposed on the pure-tone vocoder stimuli and masked by narrowband-noise modulation maskers, similar to those used in the speech masking experiment.

Based on the earlier findings, we predicted that the older listeners would exhibit poorer AM frequency selectivity and that this deficit would result in a greater effect of AM masking, particularly so at the highest masker-modulation center frequency, compared to young listeners. Specifically, we assumed that AM masking largely explains speech masking in noise (Stone and Moore, 2014) and hypothesized that differences in AM selectivity contribute to differences in speech-in-noise perception between young and older listeners (as suggested by Regev et al., 2023a, 2023b). Consequently, we expected the maskers without fluctuations to result in minimal differences in performance for young and older listeners, whereas SRTs would be poorer for older listeners when listening to speech in the presence of broadband or narrowband modulation maskers, due to a wider spread of masking across the modulation spectrum. Hence, we also expected that the differences in SRTs between young and older listener would be largest for the highest masker-modulation center frequency, as the spread of masking from the 32-Hz masker would result in more masking of the critical modulation frequencies (i.e., below 16 Hz) for the older listeners than for the young.

## 2. Methods

### 2.1. Listeners

**Fig. 1.**
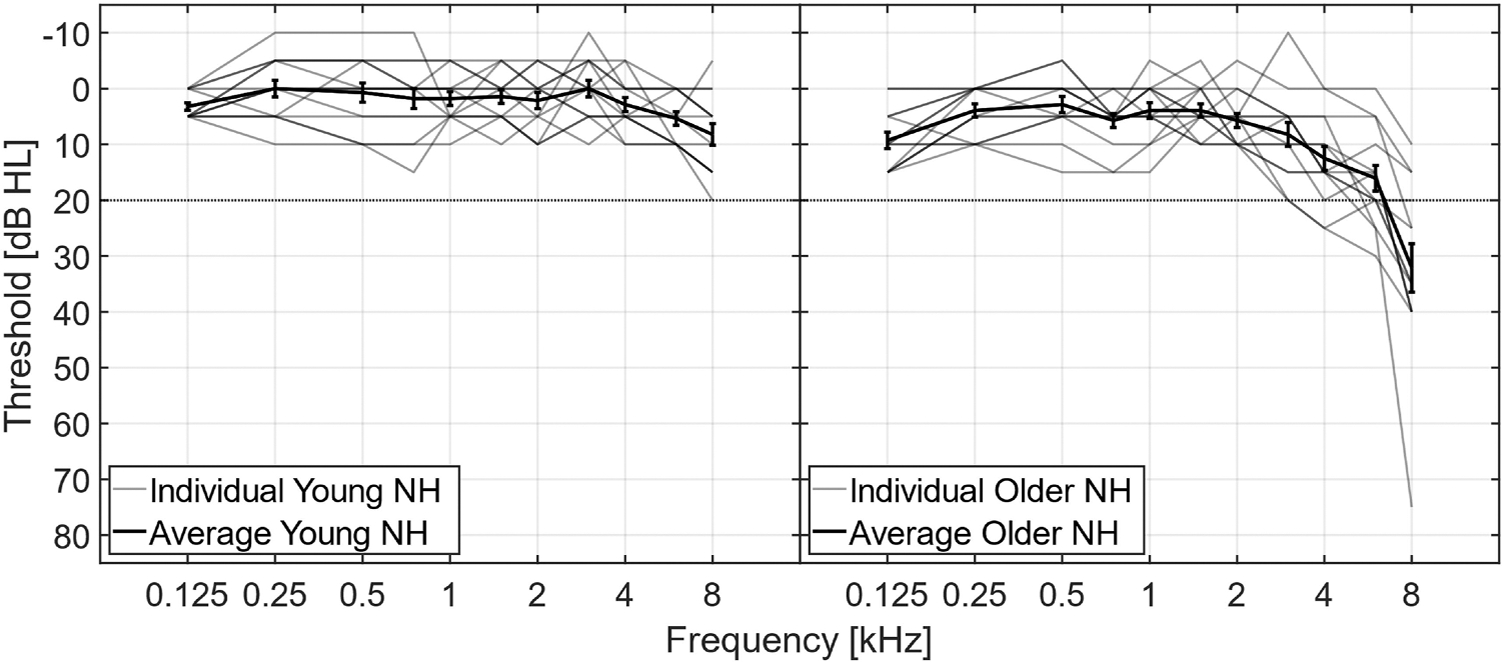
Audiometric thresholds for the test ear of each listener. The left panel shows the audiograms of the young listeners, while the right panel shows those of the older listeners. The light grey lines indicate the individual audiograms, and the black lines and error bars indicate the average and standard error across listeners. Not all the individual lines are visible due to overlap of the audiograms. The horizontal dashed line indicates the 20 dB HL criterion.

Fourteen young NH listeners, aged between 19 and 25 years (mean = 23 years, 7 female and 1 nonbinary), and fourteen older NH listeners, aged between 57 and 79 years (mean = 67 years, 10 female) took part in the study. All participants were native Danish speakers. The psychoacoustic tests were conducted monaurally, and the audiograms for the test ear of each listener are shown in Fig. 1. All young NH listeners had hearing threshold levels (HTLs) ≤ 20 dB HL at audiometric frequencies from 0.125 to 8 kHz. The older NH listeners had HTLs ≤ 20 dB HL at audiometric frequencies from 0.125 to 3 kHz, ≤ 25 dB HL at 4 kHz, ≤ 30 dB HL at 6 kHz, and ≤ 40 dB HL at 8 kHz (except for one listener with a threshold at 75 dB HL at 8 kHz). One young NH listener and three older NH listeners had previously participated in a similar AM frequency selectivity study (Regev et al., 2023a). All participants provided informed written consent and were paid for their time. Ethical approval for the study was granted by the Science Ethics Committee of the Capital Region in Denmark (reference H-16036391).

### 2.2. Procedure and apparatus

The listening tests were conducted in either single- or double-walled sound-attenuating booths. The stimuli were digitally generated using MATLAB (The Mathworks Inc., 2015), converted to analog using an RME Fireface soundcard with a resolution of 24 bits, and presented monaurally through Sennheiser HDA200 headphones. The output level of the headphones was calibrated using a GRAS RA0039 ear simulator and the frequency response of the headphones was equalized using a digital inverse filter to achieve a flat response at the eardrum. Masked-threshold patterns were collected for each listener in one session, while speech-intelligibility and AM detection data were obtained in another session. The order of testing, both between and within the two sessions, was randomized across listeners.

### 2.3. AM detection and masked-threshold patterns

Both the AM detection and masked-threshold pattern (MTP) paradigms followed a similar approach to that described by Regev et al. (2023a). The stimuli were digitally generated with a sampling frequency of 48 kHz. A three-interval three-alternative forced-choice (3I-3AFC) method was used, implemented using the AFC toolbox (Ewert, 2013). A 1-up, 2-down adaptive procedure was utilized, tracking the 70.7 % correct point on the psychometric function (Levitt, 1971). Visual feedback (“incorrect”/“correct”) was provided after each trial.

Both the AM detection and MTP tasks were conducted using a target sinusoidal modulation frequency of 4 Hz. The same CT as used in the vocoder (described below) served as the carrier. All components in the CT had the same amplitude (a flat spectrum) and a 0-degree starting phase. The carrier had a duration of 1.1 s, including 50-ms raised-cosine onset and offset ramps. The target and masker modulation (when applied) had a duration of 1 s, including 50-ms raised-cosine onset and offset ramps, and were temporally centered in the carrier. The target modulation always had a 0-degree starting phase. The intervals were separated by 500 ms of silence. The stimuli were presented at a fixed sound pressure level (SPL) of 65 dB to reduce the availability of any level- or loudness-based detection cues. The masker modulation was centered at modulation frequencies ranging from −2 to +2 octaves relative to the target modulation frequency in steps of 2/3 octave (i.e., centered at 1, 1.6, 2.5, 4, 6.3, 10.1, and 16 Hz). The masker had a fixed bandwidth of 1.4 Hz, corresponding to 1/2 octave when centered at 4 Hz. Modulation depth was expressed in dB as *M* = 20*log*_10_(*m*), where *m* is the modulation depth on a linear scale. The masker had a fixed modulation depth of −10 dB rms. For each measurement run, a 2-s segment of the masker was generated, and a random section of this masker was cut out (with replacement) and applied on each trial.

The tracking variable was the target’s modulation depth (*M*) in dB. The value of *M* started at −5 dB in the AM detection task, and at 0 dB in the MTP task. The initial step size of 4 dB was reduced to 2 dB after the first lower reversal, and to 1 dB after the second lower reversal. The threshold for each measurement run was defined as the average modulation depth of the target across six reversals obtained with the final step size. The measurement for each condition was repeated three times, and the final threshold was calculated as the average across the three repetitions. The maximum allowable modulation depth was 0 dB. If the procedure called for a modulation depth exceeding 0 dB more than three times during any measurement run, the run was aborted, and another run was added to replace it. Additionally, if the standard error across repetitions exceeded 2 dB, additional runs were conducted until this limit was met. For the AM detection task, if a consistent improvement in thresholds across repetitions was found, additional runs were conducted until the thresholds stabilized and no further decrease was observed.^1^ All listeners underwent training with one measurement run in the AM detection task and three measurement runs in the MTP task (using masker placements of −2, 0, and +2 octaves relative to the target modulation frequency).

### 2.4. Intelligibility of tone-vocoded speech

All young listeners and 13 out of 14 older listeners participated in the speech-intelligibility test, which employed the Danish Hearing in Noise Test (HINT; Nielsen and Dau, 2011). The Danish HINT uses lists comprising 20 five-word sentences spoken by a male speaker. The speech was tone-vocoded, using an approach similar to that of Oxenham and Kreft (2014). The vocoder utilized 12 channels, with center frequencies ranging from 9 to 31 Cams in steps of 2 on the ERB_N_-number scale (Glasberg and Moore, 1990), corresponding to center frequencies ranging from 375 to 6237 Hz. The channel spacing of 2 Cams was chosen to limit envelope fluctuations generated by beating between the center tones of neighboring channels. The speech was filtered into the 12 channels using high-order (1352) finite impulse response zero-phase filters. The filters had a bandwidth of 2 Cams and overlapped at the −12 dB point. Details of the channel center frequencies and edge frequencies (at the −12 dB point) are provided in Table 1. The envelope of the speech in each channel was extracted using the Hilbert transform and low-pass filtered with a 4^th^-order Butterworth filter with a 50-Hz cutoff frequency. As described by Oxenham and Kreft (2014), the 50-Hz cutoff frequency was intended to limit resolved modulation sidebands and voicing periodicity cues. Finally, the filtered envelope modulated a pure tone with a 0-degree starting phase at the center frequency of each channel, and the 12 modulated pure tones were summed to create the vocoded speech.

**Fig. 2.**
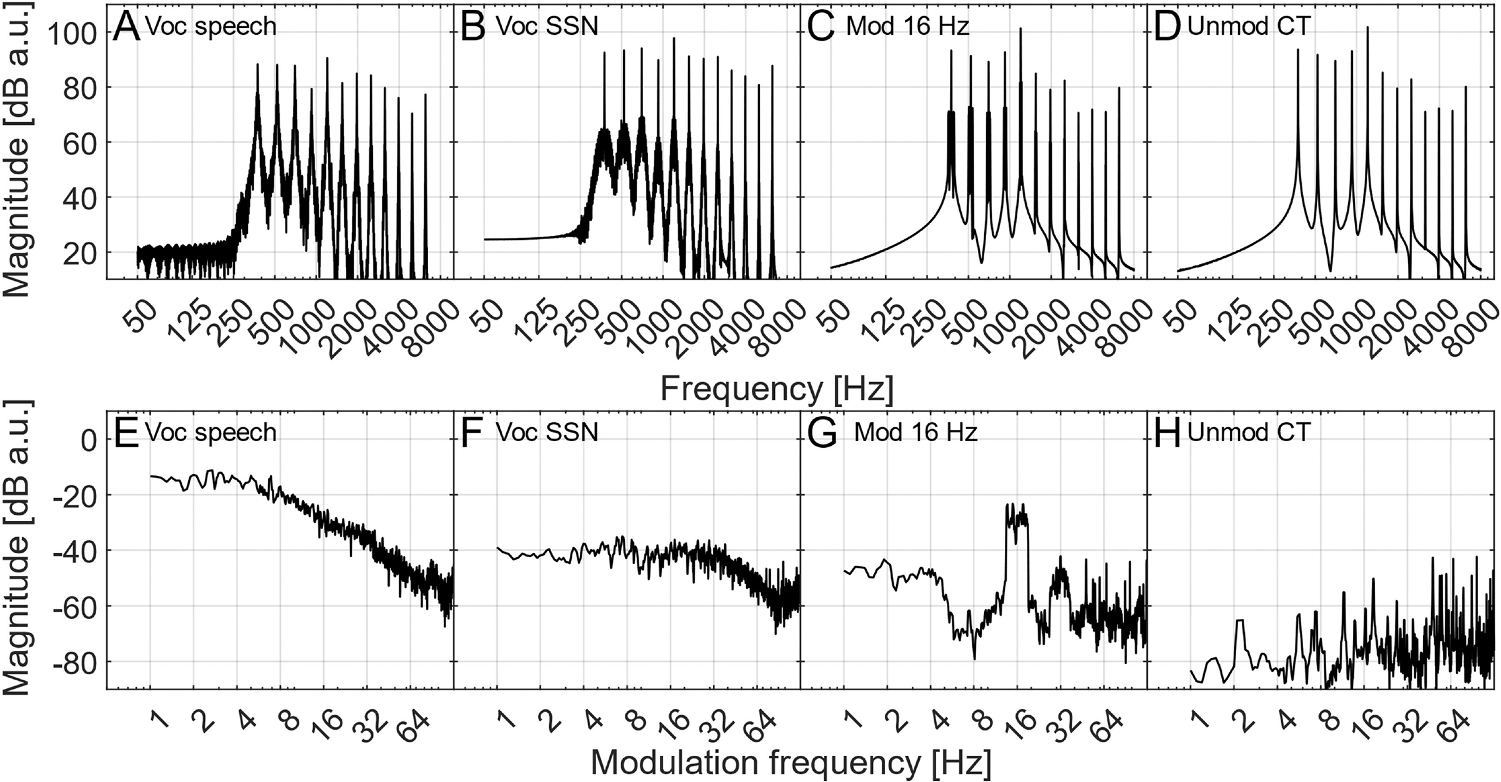
Long-term average spectra (first row) and modulation spectra (second row) for the vocoded speech (panels A and E), vocoded SSN (Voc SSN, panels B and F), an example of the modulated complex tone with a modulator centered at 16 Hz (Mod 16 Hz, panels C and G), and the unmodulated complex tone (Unmod CT, panels D and H).

Seven maskers were used: a tone-vocoded SSN, an unmodulated CT, and CTs modulated with narrow-band noise centered at 2, 4, 8, 16, and 32 Hz. The noise modulations were ½-octave wide and each carrier tone conveyed a different realization of the noise modulation to avoid comodulation across channels. The modulation depth of the noise was −10 dB rms. The component tones in the CTs had the same frequencies as for the tone vocoder, with a 90-degree starting phase. The starting phase of the masker tones led that of the target by 90 degrees so that when they were added their intensities summed (i.e., 3 dB increase at equal amplitudes) to approximate the relationship between independent sources, such as speech and noise. The amplitudes of the CTs were adjusted to match the long-term average spectrum of the vocoded speech. The long-term average spectra and modulation spectra of the vocoded speech material and maskers are shown in Fig. 2. Maskers of 25-s duration were generated and stored. The vocoded speech and masker recordings were stored separately with a sampling frequency of 44.1 kHz. The testing order of the different maskers was randomized across listeners.

SRT_50_ values were determined using one test list for each masker. In each trial, a random cut of the 25-s masker was gated on 1 s before the start of the target sentence and gated off 1 s after the end of the sentence. The vocoded speech was presented at a fixed level of 65 dB SPL, and the masker level was adjusted to achieve the desired SNR. Each trial began with a 0-dB SNR, and the SNR was modified based on the number of correctly identified words using the following rule:

(1) $$\Delta SNR=-2\left(\frac{N_{\mathrm{words}\;\mathrm{correct}}}{5}-0.5\right){SNR}_{\mathrm{step}},$$

where ΔSNR is the change in SNR, 5 is the maximum number of correct words, 0.5 represents the 50 % tracking point, and ${SNR}_{\mathrm{step}}$ is the step size. Hence, the change in SNR was negative if 3 or more words were correctly identified, and positive otherwise. The step size was 4 dB for the first 4 sentences and 2 dB afterwards. The final SRT for each masker was calculated as the average SNR across sentences 5 to 20, plus the SNR resulting from the scoring of the 20^th^ sentence. The scoring was done by a trained native Danish speaker.

Before testing, each listener underwent training on vocoded speech recognition in quiet using the HINT training lists, using a minimum of 20 sentences. If the average performance over the last 5 sentences was below 90 %, a further 5 sentences were added, and performance was reassessed. Groups of 5 sentences were added until the minimum 90 % performance criterion was reached. Only 2 young and 2 older listeners needed 25 sentences, and 1 older listener required 40 sentences.

### 2.5. Statistical analysis

Differences in AM detection thresholds between young and older listeners were analyzed using a Welch two-tailed independent-samples *t*-test. For the MTPs and speech-intelligibility data, analyses of variance (ANOVA) of linear mixed-effects model fittings were employed to assess the differences between the results for the young and older listeners. For the MTP data, the listeners were treated as a random effect, while group classification and masker-modulation center frequency were treated as fixed effects. For the speech intelligibility data, a model was initially fitted to the SRTs for all maskers to assess the main effect of testing order, which was found to be significant. Modelling the effect of order as a linear function provided a similar Akaike information criterion (AIC) value as higher-order functions, and was therefore deemed the best fit. Consequently, the linear effect of order was regressed out using a linear model, and subsequent statistical analyses were conducted on the corrected SRTs.

Two separate ANOVAs were conducted to analyze the SRTs.^2^ The first ANOVA tested the effects of age and of the presence (or absence) of broadband AM masking, including only the SRTs for the unmodulated CT and vocoded SSN maskers. The second ANOVA tested the effects of age and masker-modulation center frequency, including all maskers with imposed narrow-band noise modulation (i.e., from 2 to 32 Hz). For both analyses, the listeners were treated as a random effect, while masker type and group classification were treated as fixed effects.

For all analyses, Levene’s test was used to assess the homoskedasticity of the residuals and Shapiro-Wilk’s test was used to assess the normality of the residual distribution.^3^ Post-hoc analyses were carried out using estimated marginal means and a Holm-Bonferroni correction was applied for multiple comparisons. The level of statistical significance was set to 0.05 for all analyses.

## 3. Results

### 3.1. AM detection thresholds and masked-threshold patterns

**Fig. 3.**
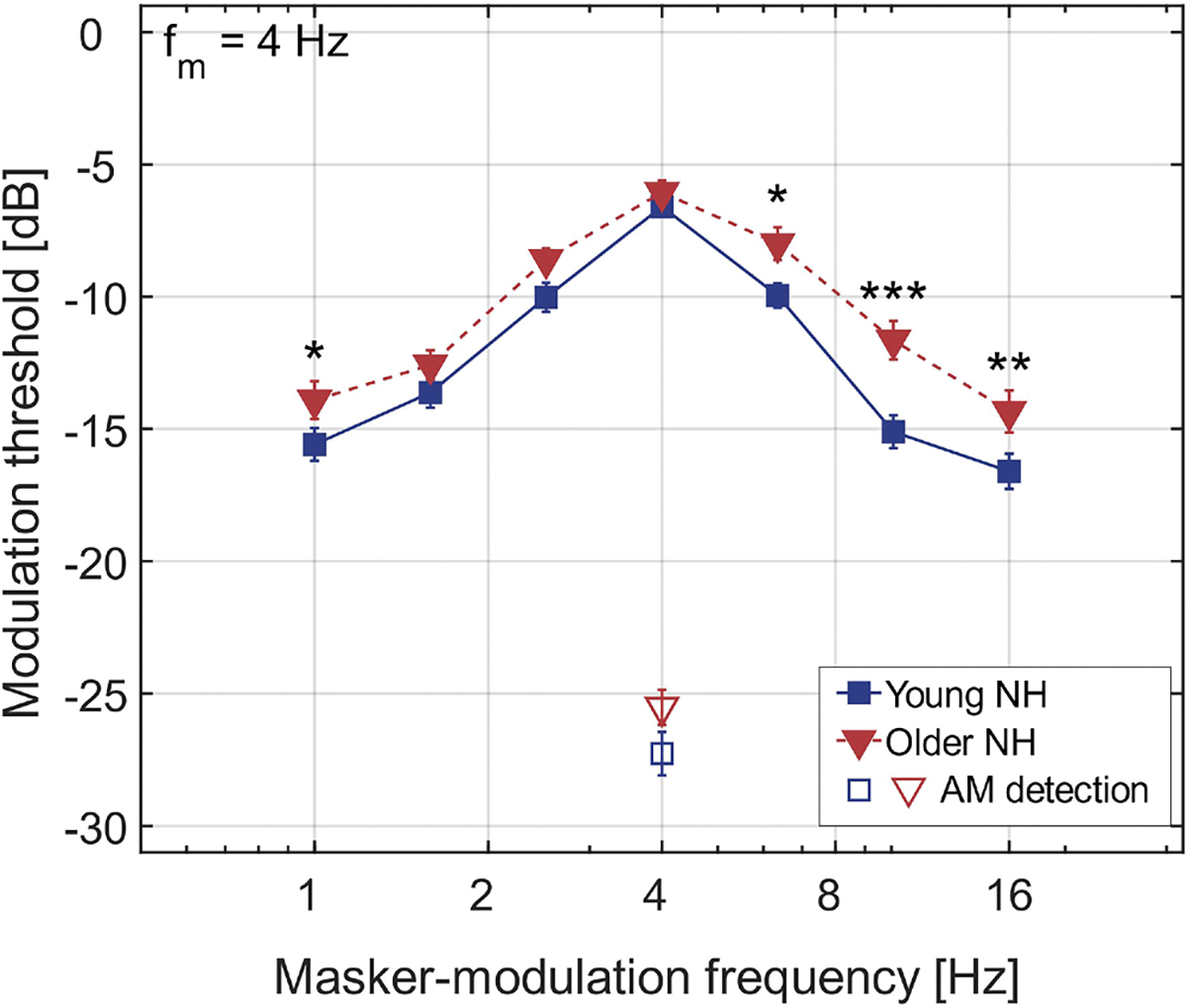
Average MTPs (closed symbols and continuous lines) and AM detection thresholds (open symbols). Results for the young listeners are depicted with blue squares, while results for the older listeners are represented by red triangles. Means and standard errors are shown. Stars denote the level of statistical significance of the differences in thresholds between the two groups. * *p* < *0.05*, ** *p* < *0.01*, *** *p* < *0.001*.

Mean AM detection thresholds (open symbols) and MTPs (closed symbols) are presented in Fig. 3, with the results for the young and older listeners shown by squares and triangles, respectively. There was no significant effect of group on the AM detection thresholds (*t*_*24.8*_ = 1.66, *p* = 0.109). In contrast, for the MTPs, significant main effects of masker-modulation center frequency (*F*_*6,156*_ = 129, *p* < 0.001) and group (*F*_*1,26*_ = 8.08, *p* = 0.009) were observed, along with a significant interaction between the two factors (*F*_*6,156*_ = 2.44, *p* = 0.028). Post-hoc analysis revealed that the older listeners had significantly higher masked thresholds than the young listeners at masker-modulation center frequencies of 1 Hz [mean difference (*M*) = 1.7 dB, standard error (*SE*) = 0.8 dB], 6.3 Hz (*M* = 2.0 dB, *SE* = 0.8 dB), 10.1 Hz (*M* = 3.5 dB, *SE* = 0.8 dB), and 16 Hz (*M* = 2.3 dB, *SE* = 0.8 dB).

To quantify this age-related broadening of the MTPs, quality (Q) factors - representing the ratio of the filter’s center frequency to its bandwidth - were derived from the group-average MTPs using the same method as Regev et al. (2024). The best-fitting linear approximation was obtained for each skirt of the MTPs, with each linear fit constrained to cross the on-target masked threshold. A Q-factor was then derived from the 3-dB-down points on the fits obtained for each skirt. The older listeners had an average Q-factor of 0.94 (mean squared error, MSE = 0.2 dB) while the young listeners had an average Q-factor of 1.21 (MSE = 0.4 dB). Individual Q-factors obtained from the listeners’ MTPs using the same approach showed very similar average results, and the difference in mean Q-factor between the two groups was significant (independent-samples *t*-test: t_25.97_ = −2.20, *p* = 0.037). The individual MTPs are shown in Supplementary Figure 1, and the group-level linear fits and individually fitted Q-factors are presented in Supplementary Figure 2.

### 3.2. Intelligibility of tone-vocoded speech

**Fig. 4.**
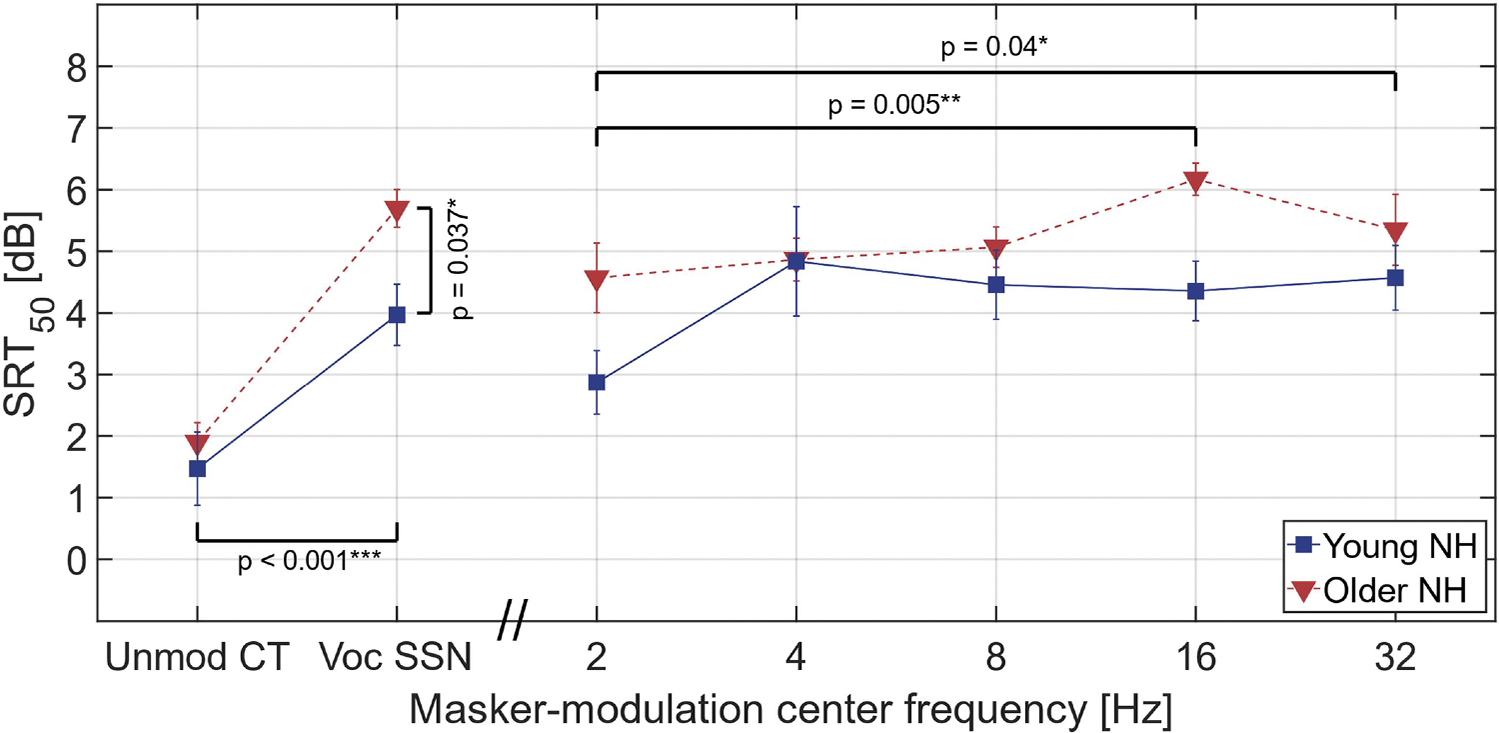
Average SRT for each masker: unmodulated CT, vocoded SSN, and modulated CTs with narrow-band noise modulators centered at 2, 4, 8, 16, and 32 Hz. Results for the young and older listeners are shown by squares and triangles, respectively. Means and standard errors are shown. The horizontal lines indicate pairwise comparisons of the SRTs for the different maskers. The vertical line indicates the main effect of age in the analysis comparing the unmodulated CT and vocoded SSN conditions. The SRTs were corrected for the effect of order and re-centered around the overall mean of the uncorrected SRTs. The p-values indicate the level of significance of the effects: *p < 0.05, **p < 0.01, ***p < 0.001.

Mean SRTs for each masker type are shown in Fig. 4, with the results for young and older listeners shown by squares and triangles, respectively. The analysis testing the effects of age and broadband AM masking (i.e., applied to the unmodulated CT and vocoded SSN maskers) revealed a significant main effect of masker type (*F*_*1,25*_ = 56.64, *p* < 0.001) and listener group (*F*_*1,25*_ = 4.85, *p* = 0.037), while the interaction was not significant (*F*_*1,25*_ = 2.41, *p* = 0.134). Post-hoc analysis showed that the unmodulated CT masker yielded significantly lower SRTs than the vocoded SSN masker (*M* = 3.2 dB, *SE* = 0.4 dB), and the older listeners had significantly higher (poorer) SRTs overall than the younger listeners (*M* = 1.1 dB, *SE* = 0.5 dB).

The analysis testing the effects of age and masker-modulation center frequency (i.e., applied to the maskers with imposed narrow-band noise modulations) revealed a significant main effect of masker-modulation center frequency (*F*_*4,100*_ = 3.71, *p* = 0.007). Although there was a tendency for the older group’s SRTs to be higher than those of the younger group, this effect did not reach significance (*F*_*1,25*_ = 3.40, *p* = 0.077). There was also no significant interaction between group and masker-modulation frequency (*F*_*4,100*_ = 1.55, *p* = 0.193). Post-hoc analysis showed that the 2-Hz modulated masker produced significantly lower SRTs than the 16-Hz masker (*M* = 1.5 dB, *SE* = 0.4 dB) and the 32-Hz masker (*M* = 1.2 dB, *SE* = 0.4 dB). There was otherwise only a small effect of modulation frequency, with SRTs remaining roughly constant for modulation frequencies between 4 and 32 Hz. Detailed results of the post-hoc analysis for pairwise comparisons of maskers with narrowband noise modulations are provided in Supplementary Table 1.

## 4. Discussion

### 4.1. Age-related reduction in AM frequency selectivity

The MTPs were broader for the older listeners than for the young listeners, while the AM detection thresholds did not differ significantly between the two groups (see Fig. 3). These findings are consistent with the results of Regev et al. (2023a) obtained under similar stimulus durations (see Fig. 7 of that study) and align with the anticipated age-related decline in AM frequency selectivity. However, the age-related broadening of the MTPs observed in the present study was less prominent, with a Q-factor ratio of approximately 1.3, compared to the ratio of 2 reported by Regev et al. (2023a). Additionally, the unmasked and masked detection thresholds in this study were lower than those reported by Regev et al. for a similar stimulus duration, the difference between studies reaching up to 4.2 dB. The greater detectability of the target AM and the reduced age-related loss of AM frequency selectivity observed in this study may be partly attributable to the use of a broadband CT carrier, in contrast to the pure-tone carrier employed by Regev et al. (2023a). Because all components of the CT carrier were comodulated in the AM detection and MTP tasks, the listeners may have gained a perceptual advantage by integrating the common AM information across the wide audio-frequency range of the stimuli (Bregman et al., 1985; Yost and Sheft, 1989; Grose et al., 2005). This increased AM sensitivity (i.e., lower unmasked AM thresholds) may have expanded the dynamic range of perceived modulations, resulting in sharper MTPs, as suggested by Wojtczak (2011).

Nevertheless, the observed age-related decline in AM selectivity supports one expected outcome of this study. Furthermore, despite the small overlap in participants between the two studies (1 young and 3 older listeners), these results provide valuable confirmation of the effect reported by Regev et al. (2023a) in a substantially different cohort and with a different carrier.

### 4.2. Effect of masker modulation frequency on speech intelligibility

The results obtained for the maskers with narrow-band noise modulation showed that the 2-Hz modulated masker yielded lower SRTs than both the 16- and 32-Hz maskers. The lower SRTs observed with the 2-Hz modulation masker are likely attributable to masking release, where listeners benefit from higher SNRs during the relatively long temporal ‘dips’ in the masker (i.e., “listening in the dips”; Buus, 1985; Cooke, 2006; Stone and Moore, 2014).

Overall, the data suggest that maskers with modulation frequencies between 4 and 32 Hz are similarly detrimental to speech intelligibility. In contrast, previous studies have indicated that modulation frequencies below 12 to 16 Hz are most critical for speech intelligibility (Drullman et al., 1994a, 1994b; Arai et al., 1999; Xu et al., 2005; Apoux and Bacon, 2008a; Elliott and Theunissen, 2009), with relatively small effects observed when modulation frequencies above 16 Hz were filtered out, (although modulation frequencies above 16 Hz have still been shown to contribute to speech intelligibility in some contexts; e.g., Shannon et al., 1995; Apoux and Bacon, 2008a). Therefore, it might have been expected that the 32-Hz modulated masker would produce lower SRTs than the other modulated maskers. However, the results showed that the 32-Hz modulated masker was as detrimental to speech intelligibility as the maskers centered at 4 to 16 Hz, suggesting no significant variation in importance across modulation frequencies.

The few existing studies that explored the role of different modulation frequencies in speech intelligibility using AM masking did report an effect of the masker’s modulation frequency content on intelligibility. Apoux and Bacon (2008b) and Stone and Moore (2014) both found that maskers conveying amplitude modulations at 4 to 16 Hz were the most detrimental to consonant identification and speech intelligibility, respectively. However, unlike the present study, which employed tone-vocoded speech, the investigations of Apoux and Bacon (2008b) and Stone and Moore (2014) used speech filtered into one or more spectral bands, where cues other than those based on the temporal envelope may have influenced their results. Additionally, only Apoux and Bacon (2008b) specified that their findings were statistically significant, and both studies reported relatively small differences in intelligibility scores across modulation frequencies. For example, Apoux and Bacon (2008b) observed up to a 9 % reduction in consonant identification, and Stone and Moore (2014) reported a 1-dB increase in SRT, which are comparable to the effects observed in the present study.

Hence, restricting access to speech modulation energy through AM masking appears to produce smaller effects on intelligibility scores compared to restricting access through filtering. However, the findings of studies employing selective modulation filtering may have been influenced by the fact that envelope information can be reconstructed from the speech fine structure (Ghitza, 2001; Atlas et al., 2004; Zeng et al., 2004; Apoux et al., 2011). Therefore, AM masking may provide a more suitable method for assessing the relative importance of different modulation frequencies in speech, as proposed by Apoux and Bacon (2008b).

One possible explanation for the lack of a modulation-frequency-specific effect in the present study, specifically the absence of a decrease in SRT for the 32-Hz modulated masker, may be that all cues for speech understanding were restricted to modulation frequencies below 50 Hz. Apoux and Bacon (2008a) suggested that the comprehension of vocoded speech might rely on all the available modulation information within each vocoder channel. If so, the listeners in the present study may have relied more on modulation information around 32 Hz than anticipated. However, the modulation power at 32 Hz in the vocoded speech was substantially reduced compared to the modulation power in the 2 to 8 Hz range (by approximately 20 dB on average, see Fig. 2E). This suggests that the 32-Hz modulation frequency region carried far less speech-relevant information, making a decrease in SRT still plausible. Conversely, as the inherent modulation power in speech declines with increasing modulation frequency above 4 Hz, the decreasing importance of these modulation frequencies may be offset by the decreasing SNR_env_ when using an equal-level masker across different modulation frequencies. These factors may have contributed to the similar effect of masker modulations between 4 and 32 Hz on the SRTs observed in the present study.

### 4.3. Effects of age and audibility on speech intelligibility

The overall effect of age on speech intelligibility was significant only in the analysis assessing the effects of broadband AM masking. A significant increase in SRTs with age is consistent with existing literature documenting age-related changes in speech intelligibility (e.g., CHABA, 1988; Pichora-Fuller and Souza, 2003; Füllgrabe et al., 2015; Goossens et al., 2017; Decruy et al., 2019; Regev et al., 2023b). Notably, although the interaction between the effects of age and masker type was not significant, there was a trend for the differences in SRTs between young and older listeners to be smaller for the unmodulated CT masker than for the vocoded SSN masker. Since the envelope power spectrum of the vocoded SSN displays a broader pattern than that of the vocoded HINT sentences (see Fig. 2E and F), the greater SRTs observed for older listeners may be related to a reduction in SNR_env_ at the output of the modulation filters resulting from a wider spread of AM masking across modulation frequencies. This trend is broadly consistent with the hypothesis that older listeners are more susceptible to AM masking of speech than young listeners, potentially due to a loss of AM selectivity.

In contrast, although there was a tendency for the older group’s SRTs to be higher than those of the younger group, there was no significant difference between young and older listeners in the pattern of SRTs across modulation frequencies. Overall, trends in the speech-intelligibility results suggest that older listeners may be more susceptible to AM masking of speech than younger listeners, although the outcomes were ambiguous, with the difference between the groups failing to reach significance in the presence of the narrow-band noise modulation maskers. This lack of significance may be attributable to the relatively low statistical power of our study. Due to time constraints, SRTs were collected from a total of 27 listeners. A power analysis indicated that 80 listeners would have been required to reliably detect the observed main effect of age with 90 % power and an alpha level of 5 %. Consequently, no definitive conclusions can be drawn from the lack of a significant age effect in cases involving narrow-band modulation maskers.

Finally, although all listeners had audiometric thresholds ≤ 25 dB HL up to 4 kHz, the older listeners’ thresholds were, on average, 7 dB higher than those of the young listeners. Even small differences in pure-tone thresholds have been shown to be associated with self-reported hearing difficulties (Brungart et al., 2025) and to affect speech intelligibility (e.g., Smoorenburg, 1992; Dubno et al., 2000), and may partially contribute to supra-threshold deficits (Léger et al., 2012). Thus, the subclinical hearing loss observed in the older listeners may have influenced the results.

### 4.4. Speech intelligibility with tone vocoders

The SRTs were high for both listener groups, with no average SRTs below 0 dB, indicating the difficulty of the speech-intelligibility task using tone-vocoded stimuli. Although the training procedure ensured that each listener achieved at least 90 % correct recognition in quiet, the introduction of a masker increased the task’s difficulty. This is further supported by the statistically significant effect of testing order observed in the speech-intelligibility data, indicating that listener performance had not fully stabilized by the end of the training session. Similarly, O’Neill et al. (2021) reported a significant effect of testing order on speech-intelligibility with tone vocoders, attributing it to long-term learning effects persisting despite a training period. Moreover, because the testing order could not be counterbalanced across conditions and listeners, it could not be fully accounted for in the statistical models. It is possible that the linear regression applied to correct for the effect of testing order did not completely eliminate its influence on the data and, consequently, on the statistical outcomes.

The task’s difficulty may be partly attributed to the use of only 12 channels in the tone vocoder. This design choice aimed to limit the amount of AM generated by beating between carrier tones in neighboring channels, ensuring that AM masking was primarily influenced by the imposed fluctuations. However, it resulted in a relatively sparse spectral representation of the stimuli. The higher performance levels observed by Oxenham and Kreft (2016) with a 16-channel tone vocoder support this notion. Their interpolated SRTs, based on psychometric functions at a speech level of 40 dB SL, were substantially lower than those measured in this study, with differences up to 6.3 dB. A more extensive training session may have mitigated the impact of the low number of channels on performance.

The number of channels in the tone-vocoder may also have influenced the pattern of SRTs across masker-modulation frequencies. Fu and Nogaki (2005) investigated the effect of the number of channels on masking release for NH listeners and cochlear-implant users using noise vocoders. They found that reducing the number of channels from 16 to 8 to 4 decreased masking release for amplitude-modulated SSN. This reduction in masking release was more pronounced for low modulation frequencies (up to ~8 Hz) than for higher frequencies (16 and 32 Hz). These findings suggest that the number of channels in the tone vocoder used in the present study may have influenced the pattern of SRTs across masker-modulation frequencies. However, Fu and Nogaki (2005) used modulated noise that was comodulated across channels, whereas the maskers in the present study were not comodulated. This absence of comodulation likely limited masking release, as indicated by the fact that all narrow-band noise modulation maskers produced SRTs comparable to those for the vocoded SSN and that only the 2-Hz modulated masker produced significantly lower SRTs than other modulated maskers (at 16 and 32 Hz). The absence of substantial masking release observed in the present study using a 12-channel tone vocoder suggests that increasing the number of channels would have had a minimal effect on masking release and, therefore, on the pattern of SRTs.

Overall, these factors underscore the importance of counter-balancing the testing order, rather than simply randomizing it, and conducting extensive training with maskers rather than in quiet for vocoder studies.

## 5. Summary and conclusions

This study aimed to investigate whether an age-related reduction in AM frequency selectivity was associated with a decline in speech intelligibility in older NH listeners, due to increased AM masking. Speech intelligibility and AM frequency selectivity were assessed for young (19–25 years old) and older (57–79 years old) NH listeners. To isolate the influence of envelope-based cues, tone-vocoded speech was presented in unmodulated and modulated maskers with varying AM frequencies. It was assumed that AM masking is the primary factor affecting speech intelligibility in this experimental paradigm. Specifically, it was expected that older listeners would exhibit a loss of AM frequency selectivity, as well as an increase in SRTs for modulated maskers and a potential modulation-frequency-specific effect of age on SRTs.

The results confirmed a reduction in AM selectivity for the older listeners, consistent with previous findings but smaller in size, likely due to the use of a different carrier signal. Additionally, SRTs obtained for maskers containing either no envelope fluctuations or broadband fluctuations showed a significant overall effect of age, and trends suggested that the increase in SRTs with age was greater in conditions with broadband AM masking than in conditions with no AM masking. However, the speech-intelligibility data for maskers with narrow-band noise modulation did not reveal a consistent effect of masker modulation frequency, and the age-related differences in SRTs for these maskers failed to reach statistical significance. Instead, the detrimental effects of AM masking on speech intelligibility were found to be broadly independent of the modulation-frequency content of the maskers across the range of 4 to 32 Hz. Overall, the question of whether older listeners are more susceptible to AM masking of speech remains unresolved. Given that the effect size appears to be small, based on the current sample, larger-scale experiments would be required to determine the interactions between age and the effects of modulation masking on speech perception.

## Supplementary Material

Supplementary Fig. 2

Supplementary Table 1

Supplementary Fig. 1

Supplementary materials

Supplementary material associated with this article can be found, in the online version, at doi:10.1016/j.heares.2025.109236.

## Figures and Tables

**Table 1:** Center frequencies and edge frequencies (at the −12 dB point) of each channel employed in the tone vocoder.

Channel	1	2	3	4	5	6	7	8	9	10	11	12

Center frequency [Hz]	375	520	700	924	1201	1545	1972	2501	3158	3973	4984	6237
Edge frequencies [Hz]	313	444	605	806	1055	1364	1747	2223	2812	3544	4451	5577
	444	605	806	1055	1364	1747	2223	2812	3544	4451	5577	6973

## Data Availability

The data for this study are available from: “Dataset for: ‘Evaluating the role of age on speech-in-noise perception based primarily on temporal envelope information’”. https://doi.org/10.11583/DTU.25809052.

## Footnotes

1 For the AM detection task, two young and four older listeners required one additional run, while one older listener required two additional runs. For the MTP task, four young and four older listeners required one additional run, one older listener required two additional runs, and one young listener required three additional runs. Additionally, two older listeners had one run aborted and replaced because the required modulation depth exceeded 0 dB.

2 An earlier version of this study applied a single ANOVA to the model fitted to the SRTs for all maskers. However, this approach combined the effects of AM masking presence (i.e., comparing the unmodulated CT and vocoded SSN conditions) and masker-modulation center frequency (i.e., for maskers with narrow-band noise modulations) into a single masker factor. To address this limitation, the analysis was revised to include two separate ANOVAs to obtain a more direct and well-defined examination of each effect separately.

3 Levene’s test was not passed for the model applied to the SRTs for maskers with narrow-band modulations. However, the deviations were minor, and the model predictions matched the average group data accurately. Therefore, no transformations of the data were applied in order to keep all analyses consistent.
