# Supplementary material for: Evaluating the role of age on speech-in-noise perception based primarily on temporal envelope information
Source: Hear Res. Author manuscript; Available in PMC 2025 Sep 17. (PMC12442876; doi:10.1016/j.heares.2025.109236)
Supplement: Supplementary Fig. 2 [file NIHMS2108181-supplement-Supplementary_Fig__2.pdf]

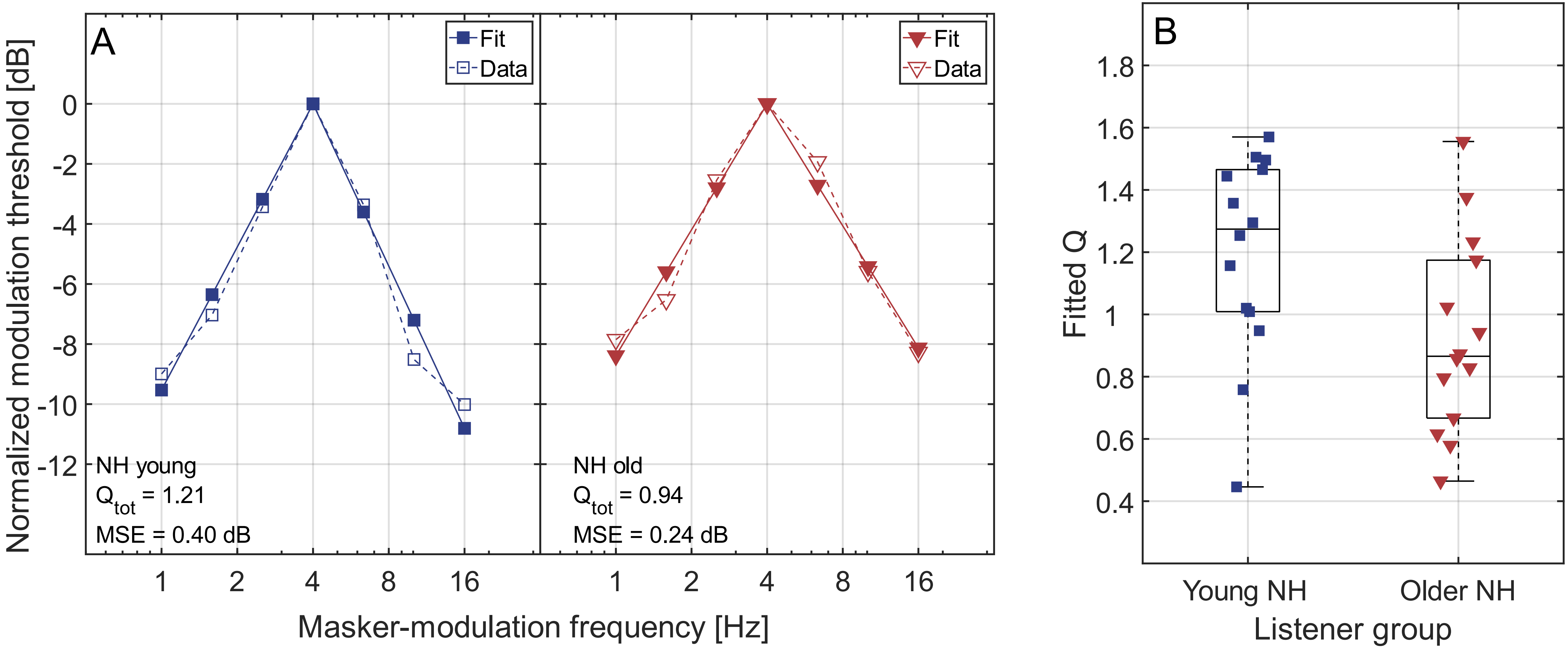

Supplementary Figure 2: **A:** Best linear fits to the group-level MTPs. The closed symbols show the fitted data and the open symbols show the experimental data. **B:** Boxplots of the Q factors fitted to the individual MTPs, for each group. Young and older listeners are shown in blue squares and red triangles, respectively.
