# Supplementary material for: Evaluating the role of age on speech-in-noise perception based primarily on temporal envelope information
Source: Hear Res. Author manuscript; Available in PMC 2025 Sep 17. (PMC12442876; doi:10.1016/j.heares.2025.109236)
Supplement: Supplementary Table 1 [file NIHMS2108181-supplement-Supplementary_Table_1.pdf]

Supplementary Table 1: Results of the post-hoc analysis on the effects of different narrow-band noise modulation maskers on speech intelligibility. The mean difference (*M*) and standard error (*SE*) are reported for each pair. Significant p-values are highlighted in bold. Note that the effect sizes are reported for the SRTs adjusted after the effect of order was accounted for.

Masker pair	*M* [dB]	*SE* [dB]	Corrected *p*-value
Mod 2 Hz – Mod 4 Hz	-1.1	0.4	0.078
Mod 2 Hz – Mod 8 Hz	-1.0		0.119
Mod 2 Hz – Mod 16 Hz	-1.5		**0.005**
Mod 2 Hz – Mod 32 Hz	-1.2		**0.043**
Mod 4 Hz – Mod 8 Hz	0.1		1
Mod 4 Hz – Mod 16 Hz	-0.4		1
Mod 4 Hz – Mod 32 Hz	-0.1		1
Mod 8 Hz – Mod 16 Hz	-0.5		1
Mod 8 Hz – Mod 32 Hz	-0.2		1
Mod 16 Hz – Mod 32 Hz	0.3		1
