# Supplementary material for: Evaluating the role of age on speech-in-noise perception based primarily on temporal envelope information
Source: Hear Res. Author manuscript; Available in PMC 2025 Sep 17. (PMC12442876; doi:10.1016/j.heares.2025.109236)
Supplement: Supplementary Fig. 1 [file NIHMS2108181-supplement-Supplementary_Fig__1.pdf]

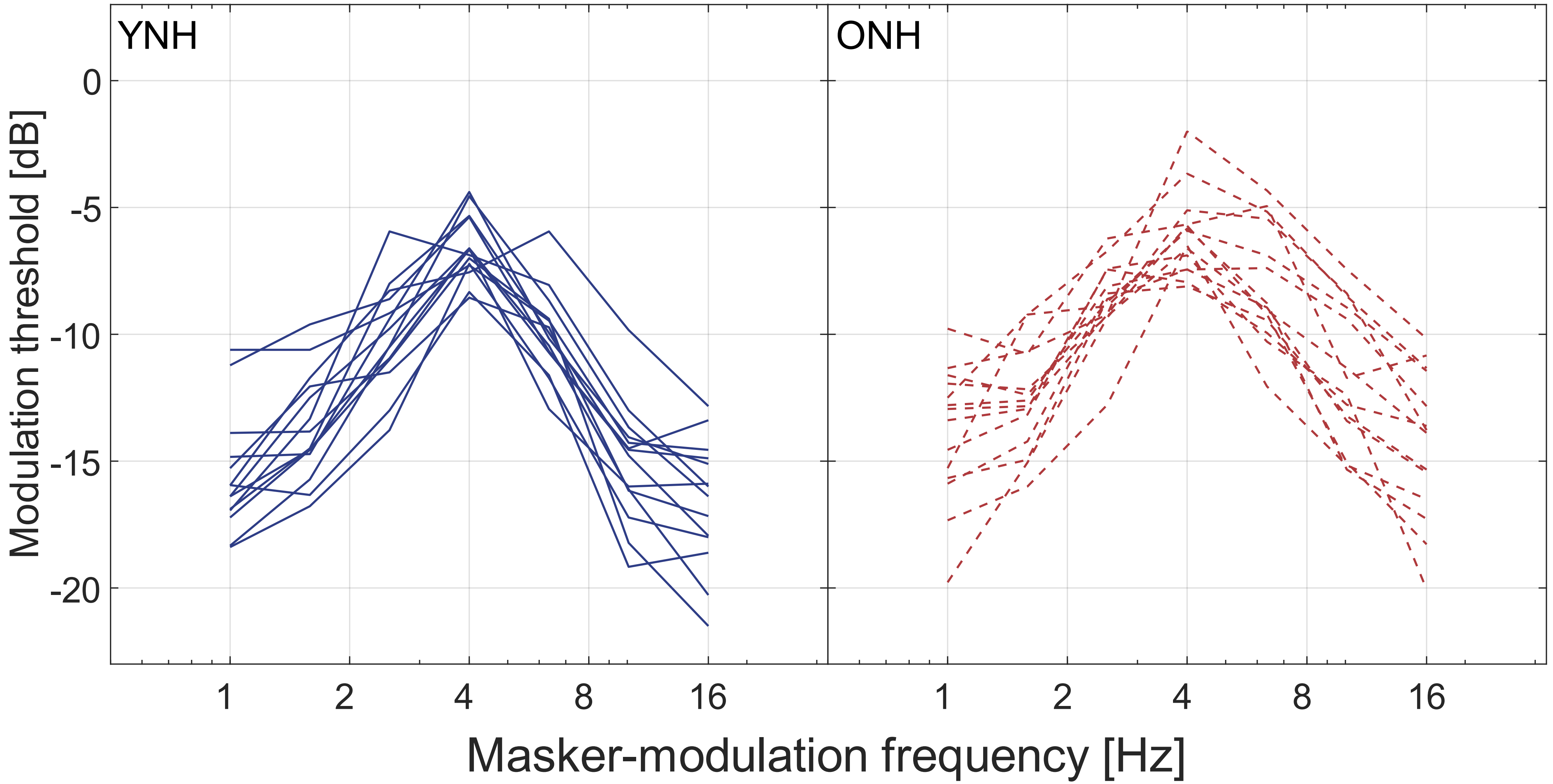

Supplementary Figure 1: Individual masked-threshold patterns. The data for young and older listeners are shown in the left and right panels, respectively.
